# Oxidative Stress and Endometriosis: A Systematic Review of the Literature

**DOI:** 10.1155/2017/7265238

**Published:** 2017-09-19

**Authors:** Gennaro Scutiero, Piergiorgio Iannone, Giulia Bernardi, Gloria Bonaccorsi, Savino Spadaro, Carlo Alberto Volta, Pantaleo Greco, Luigi Nappi

**Affiliations:** ^1^Department of Morphology, Surgery and Experimental Medicine, Section of Obstetrics and Gynecology, Azienda Ospedaliero-Universitaria S. Anna, University of Ferrara, Via Aldo Moro, 8, 44121 Cona, Ferrara, Italy; ^2^Department of Morphology, Surgery and Experimental Medicine, Section of Anaesthesia and Intensive Care University of Ferrara, Azienda Ospedaliero-Universitaria S. Anna, Via Aldo Moro, 8, 44121 Cona, Ferrara, Italy; ^3^Department of Medical and Surgical Sciences, Institute of Obstetrics and Gynecology, University of Foggia, Viale L. Pinto, 71100 Foggia, Italy

## Abstract

Endometriosis is one of the most common gynaecologic diseases in women of reproductive age. It is characterized by the presence of endometrial tissue outside the uterine cavity. The women affected suffer from pelvic pain and infertility. The complex etiology is still unclear and it is based on three main theories: retrograde menstruation, coelomic metaplasia, and induction theory. Genetics and epigenetics also play a role in the development of endometriosis. Recent studies have put the attention on the role of oxidative stress, defined as an imbalance between reactive oxygen species (ROS) and antioxidants, which may be implicated in the pathophysiology of endometriosis causing a general inflammatory response in the peritoneal cavity. Reactive oxygen species are intermediaries produced by normal oxygen metabolism and are inflammatory mediators known to modulate cell proliferation and to have deleterious effects. A systematic review was performed in order to clarify the different roles of oxidative stress and its role in the development of endometriosis. Several issues have been investigated: iron metabolism, oxidative stress markers (in the serum, peritoneal fluid, follicular fluid, peritoneal environment, ovarian cortex, and eutopic and ectopic endometrial tissue), genes involved in oxidative stress, endometriosis-associated infertility, and cancer development.

## 1. Introduction

Endometriosis is an estrogen-dependent pelvic inflammatory disease characterized by implantation and growth of endometrial tissue (glands and stroma) outside the uterine cavity [1]. It affects about 10–15% of women of reproductive age [2, 3]. The most common symptoms of the disease are pelvic pain and infertility [1]. In fact, the prevalence of endometriosis in women with pelvic pain ranges from 30 to 45% of infertile population [4]. However, endometriosis can be also asymptomatic or accompanied by symptoms such as dysmenorrhea and dyspareunia [3, 5, 6]. The etiology of endometriosis is still unclear: the implantation theory of Sampson, the coelomic metaplasia theory of Mayer, and the theory of induction are the three classic theories that have tried to designate the definitive pathogenetic mechanism of endometriosis but they have failed to establish it [7, 8]. Recent studies have addressed the role of other factors in the development of endometriotic lesions such as familiar tendency and genetic predisposition [9]. It is now widely accepted that oxidative stress, defined as an imbalance between reactive oxygen species (ROS) and antioxidants, may be implicated in the pathophysiology of endometriosis causing a general inflammatory response in the peritoneal cavity [10–12]. Reactive oxygen species are intermediaries produced by normal oxygen metabolism and are inflammatory mediators known to modulate cell proliferation and to have deleterious effects [13]. Indeed, cells have developed a wide range of antioxidant system, such as superoxide dismutase, catalase and glutathione peroxidase, and vitamin E and vitamin C, to limit ROS production, inactivate them, and repair cell damage; however, oxidative stress may occur when the balance between ROS production and antioxidant defence is disrupted [14]. Macrophages, erythrocytes, and apoptotic endometrial tissue that transplant into the peritoneal cavity through retrograde menstruation are well known inducers of oxidative stress; therefore, peritoneal production of ROS may be involved in endometriosis. Indeed, activated macrophages play an important role in the degradation of erythrocytes that release prooxidant and proinflammatory factors such as heme and iron, implicated in the formation of deleterious ROS [15].

## 2. Materials and Methods

A review of the literature was conducted in order to identify the most relevant studies reported in the English language. We searched PubMed MEDLINE electronic database (https://www.ncbi.nlm.nih.gov/pubmed) published until March 2017. The keywords used were as follows: “endometriosis,” “oxidative stress,” “oxidative stress markers,” “reactive oxygen species,” “inflammation,” and “iron.” Different combinations of the terms were used. Moreover, references in each article were searched to identify potentially missed studies.

## 3. Results and Discussion

### 3.1. Role of Iron

Recent findings have put the attention on the role of altered iron metabolism in the endometriosis development [16]. The presence of iron overload in the different components of the peritoneal cavity of endometriosis patients has been widely studied; however, it remains strongly localized in the pelvic cavity and does not affect body iron content [16].

Higher levels of iron, ferritin, and haemoglobin have been found in the peritoneal fluid of affected women than controls [17]. The stroma of endometriotic lesions and peritoneum also revealed the presence of iron conglomerates. Peritoneal iron overload may be a consequence of increased influx caused by erythrocyte degradation, resulting either from more abundant menstrual reflux or bleeding lesions or from a deficiency in the peritoneal iron metabolism system [14].

Iron metabolism by macrophages appears to be enhanced in endometriosis. In fact, siderophages, macrophages storing iron, are heavily laden with hemosiderin inside the pelvic cavity [17]. Moreover, macrophages express more transferrin receptors and are more haptoglobin-saturated [14].

Overwhelming iron can act as a catalyst in the Fenton reaction (Fe^2+^ + H_2_O_2_ → Fe^3+^ + OH^−^ + OH) to potentiate oxygen and nitrogen toxicity by the generation of a wide range of ROS, inducing oxidative injury to cells [17].

Oxidative stress is responsible for local destruction of the peritoneal mesothelium, producing adhesions for ectopic endometrial cells. Iron-binding protein haemoglobin has been identified as one of the menstrual effluent factors potentially harmful to mesothelium, leading to adhesion formation [14].

Defrere et al. showed that epithelial cells in endometriotic lesions increase the proliferative activity after erythrocyte injection in murine model, whereas desferrioxamine administration, an iron chelator, inhibits this process, suggesting that iron may contribute to endometriotic lesion growth [18].

Nuclear factor-kappa B (NF-kappa B) is a transcriptional factor that plays a role in the immune and inflammatory response. In vivo and in vitro studies have demonstrated its inflammatory activation in endometriotic cells. ROS production by iron overload induces an increase of NF-kappa B in peritoneal macrophages, leading to proinflammatory, growth, and angiogenic factors in endometriosis women than healthy controls [14].

### 3.2. Oxidative Stress Markers

The progression of endometriosis is clearly related to oxidative stress. The connection between endometriosis and the ROS production is widely accepted and deeply studied [19].

In endometriotic cells as in tumor cells, the increased production of ROS is associated with an increase in the proliferation rate [20]. For twenty years, researchers have paid attention to the oxidative stress markers in endometriotic disease. These markers studied have been collected from different samples, which can be divided in 5 main groups:

- Serum
- Peritoneal fluid
- Follicular fluid
- Ovarian cortex and endometrial tissue (ectopic and eutopic)

#### 3.2.1. Serum

Women affected by endometriosis show a higher level of oxidative stress markers than women who are not affected.

Heat shock proteins (HSPs) are intracellular proteins induced to protect cells from various insults during stress status caused by infection or inflammation. HSP70 is a stress-inducible member of HSP family. HSP70 is a chaperone protein which prevents abnormal interactions during protein synthesis. Lambrinoudaki et al. showed that women with endometriosis have a higher serum level of HSP70 [21]. The expression increase is also present in the eutopic endometrium of women affected [22].

Lipid metabolism and its connections with inflammation factors might play a role in the genesis of oxidative stress. Lipid levels have been evaluated in women with endometriosis resulting in a higher level of triglyceride, total cholesterol, and low-density lipoprotein (LDL) in serum of affected women. On the other side, lower levels of high-density lipoprotein (HDL) have been observed [23].

The increase of lipid peroxides can be considered as an oxidative stress marker. Malondialdehyde (MDA) has been evaluated as an index of lipid peroxides. Nasiri et al. observed a higher level of MDA in the serum of women with endometriosis than healthy controls [24].

Lipid peroxidation leads also to the generation of lipid hydroperoxides (LOOHs). These compounds derive from unsaturated phospholipids, glycolipids, and cholesterol. Women with endometriosis show a higher level of LOOHs than controls [23].

The concentration of vitamin E, which is a natural antioxidant, is higher in endometriosis women serum, but this finding has not been clearly explained [25, 26].

Catalase is described as an intracellular antioxidant enzyme in hepatic pathogenesis; its concentration is found to be higher in endometriosis women than healthy controls [27].

Paraoxonase-1 (PON-1) is a HDL-associated antioxidant enzyme, which is considered as a strong predictor of coronary artery disease (CAD). PON-1 shows a significant decreased activity in the serum of women with endometriosis [23, 25]. Though its activity is decreased, there is no correlation between PON-1 and the stage of the disease [28].

Another enzyme involved in oxidative stress is superoxide dismutase (SOD). SOD is an important antioxidant system. It catalyzes the dismutation of superoxide into hydrogen peroxide and oxygen. SDO shows a decreased activity in the plasma of women of endometriosis, suggesting a decreased antioxidant capacity in these women [26].

Another oxidative stress marker which appears to be diminished in the serum of women with endometriosis is 8-F2-isoprostane [25]. Thiols are molecules, which can react with oxidizing agents and mediate the formation of reversible disulphide bonds. Turgut et al. studied the antioxidant system in women with endometriosis and showed that the total antioxidant system (TAS) and the native thiol levels in the serum of women with endometriosis are significantly lower than those of controls [27, 29]. They also described higher levels of copper and ceruloplasmin in the serum of women with endometriosis, although Turkyilmaz found lower levels in contrast to the previous finding [27]. These findings support the hypothesis that low antioxidant levels are linked to the pathogenesis of endometriosis.

Andrisani et al. examined the possible involvement of carbonic anhydrase activation in response to oxidative stress in red blood cells of women with endometriosis. They found an increased enzyme activity together with a membrane increase of glutathionylated protein and a cytosolic decrease of glutathione content than control serum. The oxidation-induced activation of carbonic anhydrase is also positively correlated to glutathione content in red blood cells of women with endometriosis [30]. Most of these studies are either observational or case-control studies; moreover, measurement of biomarkers is subject to interlaboratory variations and interobserver differences. A uniform method should be used so that the results can be compared across studies.

#### 3.2.2. Peritoneal Fluid

Peritoneal oxidative stress is currently thought to be a major constituent of the endometriosis-associated inflammation. The development of peritoneal endometriotic lesions involve multiple factors based on immunological and inflammatory etiology. Peritoneal oxidative stress regulates expression of numerous genes encoding immunoregulators, cytokines, and cell adhesion molecules. Peritoneal concentration of macrophages appears to be higher in women with endometriosis and they may release prostaglandins, cytockines, growth factors, and other enzymes. It has been theorized that macrophages play an important role in the initiation, maintenance, and progression of endometriotic disease [14, 31].

Santulli et al. explored the peritoneal fluid protein oxidative status in women with endometriosis. They found higher levels of advanced oxidation protein products (AOPP) than controls. In the same way, concentrations of nitrates and nitrites are higher in affected patients than controls. Moreover, AOPP and nitrates/nitrites are higher in patients with deep infiltrating endometriosis, especially those with intestinal involvement [32]. The higher concentration of nitrates probably derives from the augmented nitric oxide (NO) activity of the peritoneal macrophages, as already described by Osborn, who observed also a higher activity of nitric oxide synthase 2 (NOS 2) [33]. Interestingly, they also observed a significant correlation between pelvic pain symptom scores and peritoneal protein oxidative stress markers in women with endometriosis [32]. Oxidative mechanisms involving LDL are higher in women with endometriosis. It consists in the oxidation of polyunsaturated fatty acid containing lipids of the lipoprotein. Murphy et al. showed higher levels of oxidized LDL (ox-LDL) than controls in peritoneal fluid [34]. Polak et al. noted that ox-LDL concentrations are higher in women with severe endometriosis [35].

MDA and LOOHs peritoneal levels are higher in women with endometriosis [36]. As a confirmation of this hypothesis, Mier-Cabrera et al. observed a decrease in the concentrations of MDA and LOOHs in women with endometriosis both in serum and peritoneal fluid after the supplementation of vitamins C and E, natural antioxidants, whose levels are low in affected women. These findings support the hypothesis of decreased antioxidant activity in peritoneal fluid of women with endometriosis [36–38]. Other oxidative stress markers found higher in peritoneal fluid of women with endometriosis are 8-hydroxy-2-deoxyguanosine, 8-isoprostane, 8-iso prostaglandin F2*α*, and 25-hydroxycholesterol [35, 39].

#### 3.2.3. Follicular Fluid

Follicular fluid (FF) plays a crucial role in the reproductive performance of oocyte. An imbalance between ROS and antioxidant systems in the FF could be responsible for abnormal oocyte development, causing DNA, cytoskeleton, and cell membrane damage, which would result in lower egg quality and endometriosis-associated infertility [24, 26].

Women with endometriosis show higher levels of lipid peroxide (LPO) and lower levels of total antioxidant capacity (TAC) than controls [24].

Singh et al. widely evaluated the FF oxidative stress of women with endometriosis. They found higher levels of ROS, MDA, and NO in the affected women FF. High concentrations of ROS and NO were found to be corresponding to immature oocytes and poor-quality embryos. The antioxidant system is less active in women with endometriosis. Antioxidant enzymatic activity, such as SOD, catalase, glutathione peroxidase, and glutathione reductase are found to be lower in studied FF. Also, nonenzymatic activity is less expressed. Vitamins A, C, and E concentration in FF of endometriosis women are significantly decreased than controls [26, 40]. Interestingly, however, the oxidative stress and antioxidant system in FF in patients with unilateral endometrioma is similar to those who do not have endometrioma [41].

#### 3.2.4. Ovarian Cortex and Endometrial Tissue

Oxidative stress induces ovarian damage. In fact, granulosa cells in patients with endometriosis show more signs of oxidative DNA damage than controls. Granulosa cells of women with endometriosis exhibit higher incidence of apoptotic bodies and nytrotyrosine than controls [42]. Ovarian cortex of women affected is also damaged by oxidative stress. Matsuzaki et al. demonstrated that ovarian cortex of women with endometriosis express higher levels of 8-hydroxy-2-deoxyguanosine than those of women with dermoid and serous cysts [43].

Oxidative stress activity and ROS levels are high in endometriosis, and their main effects on cells are translated into damage and proliferation [44]. Ngô et al. have evaluated oxidative stress level from biopsies of eutopic endometrium and endometriotic lesions. They observed a higher concentration of superoxide anions in both samples, whereas hydrogen peroxide is higher in endometriotic cells than in controls and endometrial cells. The detoxification of hydrogen peroxide is achieved through two different enzymatic systems: glutathione peroxidase and catalase. Glutathione peroxidase expression is higher in endometriotic cells than in controls, whereas catalase concentration is lower in endometriotic cells than in controls. Also SDO activity appears augmented in endometriotic lesions than healthy controls [45]. These findings show the role of oxidative stress in the control of endometriotic cell proliferation [20].

Oxidative stress plays its role through mitogen-activated protein (MAP) kinase/extracellular signal-regulated kinase (ERK) pathway for survival and proliferation of endometriotic lesions through expression and action of c-Fos and c-Jun. The ERK signalling pathway is involved in the proliferative response induced by endogenous ROS [44, 46]. Activation of ERK pathway and its connection to deep infiltrating endometriosis (DIE) is established through a specific inhibitor of phosphorylation of the protein tyrosine kinase ERK [47]. The endogenous activation of the mammalian target of rapamycin (mTOR)/AKT pathways is also involved in the development of DIE [44]. Among oxidative stress markers, 8-hydroxy-2-deoxyguanosine and MDA are higher in endometriotic lesions than healthy controls [48]. MDA levels are also positively correlated with plasma 17*β*-estradiol (E2) concentrations in the ectopic endometrioma [45]. The endometrial cells of endometriosis women subjected to both E2 and hydrogen peroxide show increased ERK phosphorylation. These results show the connection between E2 and apoptosis resistance and endometriotic lesion progression [46].

Toll-like receptors (TLR) are endogenous ligands of endometrium. TLR3 and TLR 4 are predominantly expressed in healthy endometrium and in endometriotic tissue; TLR 4 seems to promote cell growth in endometriosis [49].

### 3.3. Oxidative Stress and Endometriosis-Associated Infertility

The association between endometriosis and infertility is well established in literature. The monthly fecundity rate in infertile women with endometriosis is from 2 to 10%, whereas in healthy women, the rate is between 15 and 20% [50]. In the literature, it is well explained how ROS might affect a variety of physiologic functions such as oocyte maturation, ovarian steroidogenesis, ovulation, implantation, formation of blastocyst, luteolysis, and luteal maintenance in pregnancy. Oxidative stress affects fertility in women with endometriosis in either natural or assisted conception [11]. The imbalance between ROS and antioxidant mechanisms leads to oxidative stress status in peritoneal environment, follicular fluid, and ovary surrounding, which can partly explain the infertility status associated to endometriosis.

### 3.4. Oxidative Stress and Genes

The roles of molecular alteration such as genomic instability and cell survival are debated aspects of the pathogenesis of endometriosis. Recent genetic studies have put the attention on several elements which can be related to oxidative stress, such as cell cycle checkpoint sensors, hepatocyte nuclear factor (HNF), forkhead transcription factor (FOX), and microRNAs [51].

The role of ROS, iron, and superoxide might be an epigenetic modulation. Superoxide plays an important role in epigenetic process under physiologic and pathologic conditions and regulates main epigenetic processes of DNA methylation, histone methylation, and histone acetylation [52].

Recent studies have observed aberrant histone modifications in the promoter regions of the cell cycle checkpoint kinase genes. Thus, oxidative stress stimulates cell cycle progression and enhances cellular transformation [51].

Ito et al. suggested that iron, heme, and hemoglobin accumulation leads to oxidative stress causing DNA hypermethylation and histone modifications. DNA hypermethylation is linked to defective endometrium development in endometriosis patient [52].

HFN overexpression is linked to endometriotic cell survival probably through detoxification and antiapoptotic activated pathways [53].

FOX activity is controlled by ROS-induced posttranslational modifications. Loss of FOX disables the ability of cells to arrest at checkpoint, thereby facilitating lesion development. FOX levels are lower in endometriosis women than in controls [51].

MicroRNAs are a class of noncoding small RNAs that regulates hundreds of gene expression via both posttranslational inhibition and mRNA degradation. MicroRNAs control development, differentiation, apoptosis, proliferation, and cell survival. MicroRNAs have been studied in endometriosis with up- and downregulated levels in women affected. MicroRNA dysfunction results in immune alterations and inflammatory cytokine production [51].

Moreover, microRNAs responsible for targeting nociceptive and inflammatory molecules are downregulated in women with endometriosis, thus playing a role in the etiology of endometriotic pain [54].

Hevir et al. evaluated several genes expression involved in oxidative metabolism of estrogens. Increased expression of CYP1A1, CYP3A7, and COMT was observed in endometriosis. Expression of SULT1E1, SULT2B1, UGT2B7, NQO1, and GSTP1 was decreased. These findings exhibit a disturbed balance between phase I and II metabolizing enzymes in endometriosis, leading to excessive hydroxy-estrogen and altered ROS formation, and stimulation of ectopic endometrium proliferation [55].

### 3.5. Oxidative Stress and Endometriosis-Associated Cancer Development

Oxidative stress within endometriosis is likely to contribute to the malignant transformation process. Data from literature show at least a two-step explanation which might lead to cancer. The first step is as follows: the generation of oxidative stress-induced DNA damage evokes enhanced cell apoptosis and survival in endometriotic cells. The second step is as follows: cancer progression may be associated with persistent antioxidant production favouring a protumoral microenvironment [56]. Oxidant/antioxidant balance function is a double-edged sword, promoting cell death or carcinogenesis. Upregulation of antioxidant functions in endometriosis may result in restoration of cell survival and subsequent malignant transformation [57].

## 4. Conclusions

Reactive oxygen species have an important role in modulating many physiological functions in reproduction as well as in conditions such as endometriosis and infertility; a delicate balance exists between ROS and antioxidants in the female reproductive process that maintains redox homeostasis [58]. Oxidative stress occurs when this balance between ROS production and antioxidant defence is disrupted, and it may be due to either inadequate antioxidant protection or excess production of ROS.

Various lines of evidence support the role of oxidative stress in the development and progression of endometriosis [12, 14, 15, 59, 60]. This observation may open the way to evaluation of therapeutic approaches targeting oxidative imbalance: the oxidative stress status may represent the key to treat and, eventually, to prevent endometriosis. In particular, in the future, clinical trials will help to better clarify the efficacy of antioxidants as potential therapies of endometriosis.
